# Emergency department presentations by trans and gender diverse people in Sydney, Australia: Retrospective case series

**DOI:** 10.1111/1742-6723.70031

**Published:** 2025-03-19

**Authors:** Emily Symes, Naomi Derrick, Thomas Hicks, Rhys Ross‐Browne, Louisa Degenhardt, Rachel Sutherland, Radhika Seimon, Michael Dinh

**Affiliations:** ^1^ National Drug and Alcohol Research Centre University of New South Wales Sydney New South Wales Australia; ^2^ Green Light Institute, Royal Prince Alfred Hospital Sydney New South Wales Australia; ^3^ Drug Health Service Royal Prince Alfred Hospital Sydney New South Wales Australia; ^4^ Emergency Department Canterbury Hospital Sydney New South Wales Australia; ^5^ Emergency Department Royal Prince Alfred Hospital Sydney New South Wales Australia; ^6^ Susan Wakil School of Nursing and Midwifery University of Sydney Sydney New South Wales Australia; ^7^ Emergency Department Camden and Campbelltown Hospitals Sydney New South Wales Australia

**Keywords:** electronic health record, emergency medicine, gender‐nonconforming person, mental health, transgender person

## Abstract

**Objective:**

Comprehensively describe patient and presentation characteristics of trans and gender diverse (TGD) people attending the ED.

**Methods:**

Retrospective case series that evaluated TGD people of all ages presenting to a tertiary, inner‐city ED in Sydney, New South Wales, over a 5‐year period. TGD people were identified using the ED patient tracking system, triage text and clinical notes in the electronic medical records (eMR). Patient and presentation data were extracted and descriptively analysed, including clinical characteristics, mismatches in registered gender and name, and use of non‐affirming language in discharge letters.

**Results:**

A total of 340 TGD patients with 1519 ED presentations were identified. The number of ED presentations per year by TGD people increased by 74.2% over 5 years. Presentations were prioritised Australasian Triage Scale category 1–3 in 76.7%. Hospital admission was required in 25.5%, and 8.7% left prior to treatment completion. Suicidal ideation was the most common presenting problem (13.8%) and mental health was the most common ED diagnostic category (29.4%). The gender and name registered in the eMR correctly matched the patient's current identity in 47.1% and 56.8%, respectively. Misgendering and/or deadnaming occurred in 22.6% of those receiving an ED discharge letter.

**Conclusion:**

Most TGD people identified by the present study had high acuity ED presentations, often presenting with acute mental health problems, and one‐quarter were subsequently admitted to hospital. Mismatched patient details and misgendering and/or deadnaming on discharge letters were common. These findings highlight clear opportunities to improve the care of TGD people in the ED.

## Graphical abstract

Trans and gender diverse people identified by our study had elevated rates of high‐acuity presentations, mental health‐related presentations, hospital admissions and leaving prior to treatment completion. Mismatches of patient name and gender registered in the electronic medical record were common, as were misgendering and deadnaming.

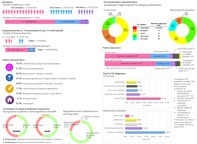

**Key findings**

- Trans and gender diverse (TGD) people identified by our study had elevated rates of high‐acuity presentations, mental health‐related presentations, hospital admissions, and leaving prior to treatment completion.
- Mismatches of patient name and gender registered in the electronic medical record (eMR) were common, as were misgendering and deadnaming – referring to an individual according to their former gender or name, respectively – in ED discharge letters.
- The current eMR system does not adequately identify or describe TGD people, and we developed a novel ascertainment method to identify TGD people presenting to the ED.

## Introduction

Trans and gender diverse (TGD) people are a minority population with significant health and social inequalities. Compared to the cisgender population, they experience elevated rates of mental and physical ill health,^1^, ^2^ and substance use.^3^ These disparities may arise from persistent exposure to socially based minority stressors, including discrimination, harassment and non‐affirmation of one's gender identity.^4^ Chronically elevated levels of minority stress have been associated with worse health outcomes in TGD people.^5^, ^6^

TGD people often encounter multiple barriers when accessing healthcare, including discrimination, unknowledgeable clinicians, unnuanced communication and service denial,^7^, ^8^ which may contribute to negative healthcare experiences.^9^ Healthcare avoidance is also common and may be attributed to fears of intolerance and mistreatment, prior healthcare dissatisfaction, and a health system that is non‐inclusive towards TGD people.^8^ Several community surveys of TGD people with a prior ED presentation have shown that approximately one‐half reported a TGD‐specific negative ED experience and 21%–49% have avoided attending the ED when care was needed.^7^, ^10^

The existing literature exploring the ED care of TGD people is predominantly qualitative, focusing on the experiences of TGD patients attending the ED^11^, ^12^ or evaluating the knowledge and training of emergency healthcare workers.^13^ There are few studies quantitatively assessing the characteristics of TGD people attending the ED. These studies demonstrate a higher likelihood of mental health presentations^14^, ^15^ and hospital admission^14^, ^15^, ^16^ compared to non‐TGD people. However, they are limited by the use of low‐quality ascertainment methods to identify TGD people or evaluate a subset of all TGD people attending the ED.^14^, ^17^ Our study uses a novel approach to identify TGD people presenting to the ED and aims to comprehensively evaluate patient and presentation characteristics of this group, including mismatches in registered gender and name, and the use of non‐affirming language in discharge letters.

## Methods

### Design, patient population and setting

This was a retrospective case series evaluating TGD people of all ages presenting to a tertiary, inner city ED in Sydney, New South Wales (NSW), Australia, over a 5‐year period. There are over 80 000 presentations to this ED annually, and it provides care to one of the largest Lesbian, Gay, Bisexual, TGD, Intersex and Queer (LGBTIQ+) communities in Australia.^18^

### Identification of TGD people

All ED presentations between 1 November 2018 to 31 October 2023, were identified from the ED patient tracking system (Cerner FirstNet) using the inbuilt data extraction tool, Discern Analytics 2.0. Patient and presentation information were obtained using this tool (Appendix S1).

Patients that may be TGD were screened using three approaches: search of triage text using keywords that may indicate a TGD identity (Appendix S2), gender recorded as ‘Indeterminate’ or ‘Unknown’, and name included variations of ‘prefer’. The electronic medical records (eMR) of patients fulfilling the screening criteria were reviewed by a primary extractor (ES) and double‐checked by two independent secondary extractors (ND, TH) to determine whether a patient was TGD. Patients were confirmed to be TGD if this was explicitly documented in their eMR, they received gender‐affirming therapy (hormonal and/or surgical), or their registered gender in the eMR changed over time. Although a patient's gender and name may be overwritten in their eMR profile, their previous details are still found in the headers of all clinical documentation prior to this change, including discharge letters.

**Figure 1:**
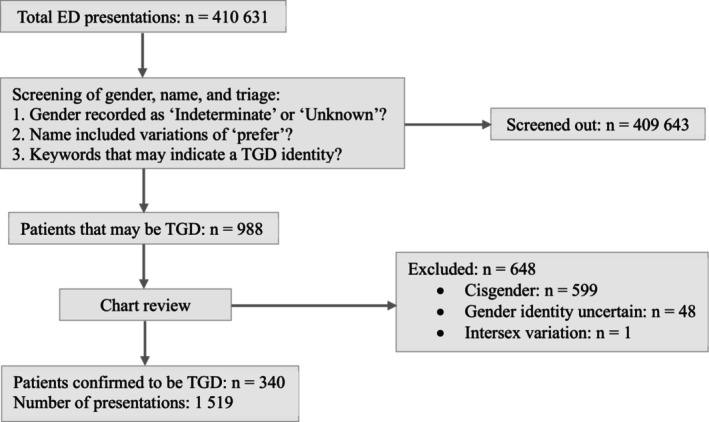
Screening and inclusion/exclusion of trans and gender diverse patients presenting to an ED in Sydney, Australia.

Patients were excluded if they were cisgender, prescribed hormone therapy for reasons other than gender affirmation, or had an intersex variation (Fig. 1). Conflicts were resolved through discussion between two authors who are TGD and emergency physicians (ES, RR‐B).

For each included patient, all ED presentations within the 5‐year study period were evaluated through a comprehensive chart review. Extraction of clinical information was performed by the primary extractor and double‐checked by two independent secondary extractors. Conflicts were resolved through discussion with a third adjudicator (RR‐B).

### Patient characteristics

Sociodemographic characteristics were collected for each patient, including age, gender identity, housing and employment. Clinical characteristics, such as lifetime diagnoses of mental health and neurodevelopmental disorders, recent (past 12 months) use of specific substances, lifetime substance dependence and lifetime recreational injecting drug use were recorded. ED frequent presenters, defined as presenting 10 or more times in any 365‐day period,^19^ were identified. Discrepancies in a patient's gender and name currently registered in the eMR, and obtained through chart review were documented. All patient characteristics were anchored from a patient's most recent ED presentation, except for age, which was listed from their earliest presentation within the study period. Information from all prior encounters was used to supplement incomplete sociodemographic and clinical data.

### Presentation characteristics

The characteristics of each patient's ED presentations were also recorded, including Australasian Triage Scale (ATS) category, ED length of stay, disposition and re‐presentation to any of the four EDs in Sydney Local Health District (SLHD) within 30 days of hospital discharge. ED discharge letters were reviewed to determine whether non‐affirming language was used, specifically *misgendering* and *deadnaming* – referring to an individual by their former gender or name, respectively.^20^ To evaluate this, the gender and name written by the clinician in the body of the discharge letter were compared with the gender and name documented in the clinical notes at that time. The auto‐filled demographic details located in the discharge letter header were not used for comparison, as these were populated from the patient information registered in the eMR and not under clinician control. ED discharge letters were not generated if a patient was admitted to hospital, did not wait for care to commence following triage, or left prior to treatment completion. Finally, the reasons for presentation were extracted, including presenting problem and ED diagnosis, which were categorised according to a previous study by Dinh and colleagues.^21^

### Data analysis

Patient and presentation characteristics were disaggregated according to three gender identities: trans female, trans male and non‐binary. Descriptive analysis was performed using SAS version 8.3.

### Ethics

The present study was approved by the SLHD human research ethics committee (ETH 02844).

## Results

There were 410 631 ED presentations to the hospital over the 5‐year study period. Screening identified 988 unique patients who may be TGD. Exclusion of 648 patients occurred: 599 were cisgender, 48 had an uncertain gender identity and one had an intersex variation. A final population of 340 TGD patients with 1519 presentations was included (Fig. 1).

### Patient characteristics

Disaggregation according to TGD identity revealed 172 (50.6%) trans females, 106 (31.2%) trans males, and 62 (18.2%) non‐binary people (Table 1). The median age of this population was 26 (interquartile range 20.5–34.0) years. The gender and name registered in the eMR correctly matched the patient's current gender identity and name in 47.1% and 56.8% of people, respectively. No non‐binary patients had a correctly matched gender.

**TABLE 1:** Sociodemographic and clinical characteristics of trans and gender diverse (TGD) patients presenting to an ED in Sydney, Australia, separated by TGD identity

	Total, *n* (%)	Trans female, *n* (%)	Trans male, *n* (%)	Non‐binary, *n* (%)
Number	340 (100.0)	172 (50.6)	106 (31.2)	62 (18.2)
Demographics				
Age (IQR), years	26 (20.5–34)	28 (22–39.5)	24 (18–29)	23.5 (18–28)
Congruence of gender				
Yes	160 (47.1)	108 (62.8)	52 (49.1)	0 (0.0)
No	180 (52.9)	64 (37.2)	54 (50.9)	62 (100.0)
Congruence of name				
Yes	193 (56.8)	102 (59.3)	61 (57.5)	30 (48.4)
No	119 (35.0)	56 (32.6)	41 (38.7)	22 (35.5)
Uncertain	28 (8.2)	14 (8.1)	4 (3.8)	10 (16.1)
Housing				
Group home or supported accommodation	11 (3.2)	5 (2.9)	3 (3.8)	3 (4.8)
Lives alone	77 (22.6)	49 (28.5)	15 (14.2)	13 (21.0)
Lives with others (e.g. family, friends)	178 (52.4)	73 (42.4)	71 (67.0)	34 (54.8)
Unstable housing†	31 (9.1)	23 (13.4)	3 (2.8)	5 (8.1)
Not assessed or unknown	43 (12.6)	22 (12.8)	14 (13.2)	7 (11.3)
Employment				
Employed	122 (35.9)	52 (30.2)	45 (42.5)	25 (40.3)
Student	64 (18.8)	19 (11.0)	31 (29.2)	14 (22.6)
Unemployed	112 (32.9)	74 (43.0)	21 (19.8)	17 (27.4)
Not assessed or unknown	42 (12.4)	27 (15.7)	9 (8.5)	6 (9.7)
Regular GP	261 (76.8)	121 (70.3)	93 (87.7)	47 (75.8)
ED frequent presenter‡	19 (5.6)	13 (7.6)	6 (5.7)	0 (0.0)
Mental health and neurodevelopmental diagnoses				
Lifetime mental health and neurodevelopmental diagnoses				
Any mental health problem	260 (76.5)	124 (72.1)	82 (77.4)	54 (87.1)
Anxiety disorders	155 (45.6)	71 (41.3)	50 (47.2)	34 (54.8)
Neurodevelopmental disorders (e.g. ADHD, ASD)	92 (27.1)	31 (18.0)	36 (34.0)	25 (40.3)
Mood disorders (e.g. depression, bipolar)	196 (57.6)	91 (52.9)	61 (57.6)	44 (71.0)
Other mental health diagnoses§	52 (15.3)	24 (14.0)	16 (15.1)	12 (19.4)
Suicide attempt	95 (27.9)	50 (29.1)	24 (22.6)	21 (33.9)
Trauma and stressor disorders (e.g. PTSD, cPTSD)	87 (25.6)	39 (22.7)	23 (21.7)	25 (40.3)
Substance use				
Recent¶ substance use				
Alcohol	206 (60.6)	106 (61.6)	60 (56.6)	40 (64.5)
Smoking cigarettes/vaping nicotine	128 (37.6)	74 (43.0)	27 (25.5)	27 (43.5)
Any illicit substance†† use	149 (43.8)	72 (41.9)	41 (38.7)	36 (58.1)
Cannabis	104 (30.6)	42 (24.4)	34 (32.1)	28 (45.2)
Methamphetamine	41 (12.1)	28 (16.3)	5 (4.7)	8 (12.9)
Other substances‡‡	63 (18.5)	34 (19.8)	13 (12.3)	16 (25.8)
Lifetime substance dependence§§	61 (17.9)	41 (23.8)	11 (10.4)	9 (14.5)
Lifetime recreational injecting drug use¶¶	37 (10.9)	29 (16.9)	4 (3.8)	4 (6.5)

^**†**^ Unstable housing: refers to experiencing primary homelessness (sleeping rough or living in an improvised shelter, such as a car or tent), secondary homelessness (moving between temporary shelters, such as crisis accommodation or ‘couch surfing’), or tertiary homelessness (living in a shelter that is below minimum community standards, such as a boarding house).^22^

^‡^ ED frequent presenter: presenting ≥10 times in any 365‐day period.^19^

^§^ Other mental health diagnoses include dissociative disorders, feeding or eating disorders, obsessive‐compulsive disorders, schizophrenia or primary psychotic disorders, and somatic symptom disorder.

^¶^ Recent substance use: within the past 12 months of the patient's most recent ED presentation.

^††^ Illicit substance: ‘any drug which is illegal to possess or use, as well as any legal drug used in an illegal manner’^23^ including cannabis.

^‡‡^ Other substances: includes 3,4‐methylenedioxymethamphetamine (MDMA), non‐medical use of benzodiazepines, cocaine, gamma‐hydroxybutyrate (GHB) and analogues, heroin, ketamine, non‐medical use of opioids and lysergic acid diethylamide (LSD).

^§§^ Substance dependence: documented history of substance dependence or has received treatment for their substance use.

^¶¶^ Injecting drug use (IDU): excludes injection of gender‐affirming hormone therapy or cosmetics.

Three quarters (76.5%) of TGD patients reported lifetime mental health or neurodevelopmental disorder diagnoses. The most reported diagnoses were mood disorders (57.6%), anxiety disorders (45.6%) and previous suicide attempts (27.9%).

### Presentation characteristics

The 1519 ED presentations by TGD people equate to 0.37% of all presentations during the 5‐year study period. There were year‐on‐year increases in the proportion of total ED presentations by TGD people, with a 74.2% increase over 5 years (Table 2).

**TABLE 2:** Presentations per study year among trans and gender diverse patients presenting to an ED in Sydney, Australia, separated by TGD identity

Study year	Total ED presentations (*n*)	ED presentations by TGD people (*n*)	Proportion of total (%)	Trans female, *n* (%)	Trans male, *n* (%)	Non‐binary, *n* (%)
1 November 2018 to 31 October 2019	83 721	213	0.25	137 (0.16)	62 (0.07)	15 (0.02)
1 November 2019 to 31 October 2020	80 031	267	0.33	148 (0.18)	93 (0.12)	26 (0.03)
1 November 2020 to 31 October 2021	78 807	317	0.40	201 (0.26)	80 (0.10)	36 (0.05)
1 November 2021 to 31 October 2022	83 289	351	0.42	215 (0.26)	96 (0.12)	40 (0.05)
1 November 2022 to 31 October 2023	84 783	371	0.44	203 (0.24)	99 (0.12)	69 (0.08)
Totals	410 631	1519	0.37	904 (0.22)	429 (0.10)	186 (0.05)

Trans females accounted for the greatest number of ED presentations. Specifically, 172 trans females presented 904 times (59.5% of all TGD ED presentations), 102 trans males presented 429 times (28.2%) and 62 non‐binary people presented 186 times (12.2%; Table 3). Regarding ED frequent presenters, 13 trans females presented 417 times and six trans males presented 127 times, accounting for 46.1% and 29.6% of presentations for these TGD identities.

**TABLE 3:** Presentation characteristics of trans and gender diverse (TGD) patients presenting to an ED in Sydney, Australia, separated by TGD identity, over a 5‐year period from November 2018 to October 2023

	Total, *n* (%)	Trans female, *n* (%)	Trans male, *n* (%)	Non‐binary, *n* (%)
Number of presentations				
Number of presentations	1519 (100.0)	904 (59.5)	429 (28.2)	186 (12.2)
Presentations per patient†	2 (1–4)	2 (1–5)	2 (1–5)	2 (1–4)
ED frequent‡ presenters				
Number of presentations	544 (35.8)	417 (46.1)	127 (29.6)	0 (0.0)
Presentations per patient	28.6	32.1	21.2	0
After hours presentations§	1084 (71.4)	638 (70.6)	313 (73.0)	133 (71.5)
Characteristics of presentations				
Australasian Triage Scale (ATS) category				
1 (Resuscitation)	6 (0.4)	5 (0.6)	1 (0.2)	0 (0.0)
2 (Emergency)	404 (26.6)	252 (27.9)	103 (24.0)	49 (26.3)
3 (Urgent)	755 (49.7)	432 (47.8)	226 (52.7)	97 (52.2)
4 (Semi‐urgent)	316 (20.8)	189 (20.9)	89 (20.8)	38 (20.4)
5 (Non‐urgent)	38 (2.5)	26 (2.9)	10 (2.3)	2 (1.1)
ED Length of stay				
Median (IQR) (min)	263 (173–525)	242 (167–528.5)	250 (178–498)	304 (182–669)
Disposition				
Admitted	388 (25.5)	246 (27.2)	100 (23.3)	42 (22.6)
Did not wait	64 (4.2)	46 (5.1)	11 (2.6)	7 (3.8)
Leaving prior to treatment completion	132 (8.7)	97 (10.7)	24 (5.6)	11 (5.9)
Transferred to another hospital	31 (2.0)	14 (1.6)	13 (3.0)	4 (2.2)
Treatment complete	904 (59.5)	501 (55.4)	281 (65.5)	122 (65.6)
Discharge documents				
Discharge document provided	765 (50.4)	417 (46.1)	242 (56.4)	106 (57.0)
Not misgendered and/or deadnamed	584 (76.3)	332 (79.6)	188 (77.7)	64 (60.4)
Misgendered and/or deadnamed	173 (22.6)	83 (19.9)	52 (21.5)	38 (35.8)
Unclear or unknown	8 (1.0)	2 (0.5)	2 (0.8)	4 (3.8)
Representation within 30 days	641 (42.2)	452 (50.0)	147 (34.3)	42 (22.6)

† Median (interquartile range).

‡ ED frequent presenter: presenting ≥10 times in any 365‐day period.^19^

§ After‐hours presentations: attending the ED outside of 8.00–17.00 hours, Monday to Friday.

ED presentations were designated ATS category 1 (resuscitation) in 0.4% (*n* = 6), category 2 (emergency) in 26.6% (*n* = 404), category 3 (urgent) in 49.7% (*n* = 755), category 4 (semi‐urgent) in 20.8% (*n* = 316) and category 5 (non‐urgent) in 2.5% (*n* = 35). Hospital admission was required in 25.5% of presentations. The proportion of presentations that did not wait or left prior to treatment completion was 4.2% and 8.7%, respectively.

An ED discharge letter was generated in 50.4% of presentations. Misgendering and/or deadnaming occurred in 22.6% of generated discharge letters.

Suicidal ideation was the most common presenting problem (13.8%), followed by abdominal pain (8.0%) and chest pain (7.3%) (Table 4). The most common ED diagnostic category was mental health in 29.4% of presentations, abdominal/gastrointestinal in 11.7% and injury in 9.7%.

**TABLE 4:** Reasons for presentations among trans and gender diverse (TGD) patients presenting to an ED in Sydney, Australia, separated by TGD identity

	Total (*n* = 1519), *n* (%)	Trans female (*n* = 904), *n* (%)	Trans male (*n* = 429), *n* (%)	Non‐binary (*n* = 186), *n* (%)
Presenting problem				
MH – suicidal ideation	210 (13.8)	136 (15.0)	42 (9.8)	32 (17.2)
Pain, abdominal	121 (8.0)	66 (7.3)	41 (9.6)	14 (7.5)
Pain, chest	111 (7.3)	67 (7.4)	31 (7.2)	13 (7.0)
Care – patient review	89 (5.9)	61 (6.7)	26 (6.1)	2 (1.1)
MH – self harm	88 (5.8)	44 (4.9)	36 (8.4)	8 (4.3)
MH – overdose intentional	67 (4.4)	37 (4.1)	20 (4.7)	10 (5.4)
MH – mental health problem	49 (3.2)	31 (3.4)	11 (2.6)	7 (3.8)
MH – altered mental status	44 (2.9)	22 (2.4)	12 (2.8)	10 (5.4)
MH – behavioural disturbance	41 (2.7)	27 (3.0)	7 (1.6)	7 (3.8)
Unwell	40 (2.6)	24 (2.7)	15 (3.5)	1 (0.5)
ED diagnosis				
Mental health	446 (29.4)	266 (29.4)	111 (25.9)	69 (37.1)
Abdominal/gastrointestinal	177 (11.7)	103 (11.4)	56 (13.1)	18 (9.7)
Injury	148 (9.7)	74 (8.2)	57 (13.3)	17 (9.1)
Drug and alcohol/toxicology	142 (9.3)	94 (10.4)	32 (7.5)	16 (8.6)
Cardiovascular	128 (8.4)	71 (7.9)	40 (9.3)	17 (9.1)
Additional factors involved with presentation				
Substance‐involved†	282 (18.6)	211 (23.3)	40 (9.3)	31 (16.7)
Complication of gender‐ affirming therapy	8 (0.5)	3 (0.3)	3 (0.7)	2 (1.1)
Specific substances involved with presentation				
Alcohol	184 (12.1)	139 (15.4)	31 (7.2)	14 (7.5)
Benzodiazepines	19 (1.3)	15 (1.7)	4 (0.9)	0 (0.0)
Cannabis	42 (2.8)	22 (2.4)	3 (0.7)	17 (9.1)
Meth/amphetamine	59 (3.9)	48 (5.3)	4 (0.9)	7 (3.8)
Other substances‡	43 (2.8)	37 (4.1)	2 (0.5)	4 (2.2)

† Substance‐involved: includes intoxication, accidental overdose, withdrawal, medical complications, or psychiatric complications.

‡ Other substances: includes 3,4‐methylenedioxymethamphetamine (MDMA), non‐medical use of benzodiazepines, cocaine, gamma‐hydroxybutyrate (GHB) and analogues, heroin, ketamine, non‐medical use of opioids and lysergic acid diethylamide (LSD).

## Discussion

Our study utilised a novel approach to identify TGD people presenting to the ED. It is the first to evaluate specific ED presentation characteristics of TGD people and quantify mismatches of gender and name in the eMR, as well as misgendering and deadnaming in ED discharge letters.

We identified 340 TGD patients with 1519 ED presentations across the 5‐year study period, representing 0.37% of all ED presentations. Although our method likely under‐reports all TGD people presenting to this ED, this was much higher than the proportion reported in a US study that identified TGD people from a national ED database using ICD diagnostic codes related to gender incongruence (0.001%–0.016%).^15^ The use of these ICD codes inadequately identifies TGD people from administrative data, detecting only 20.9–21.6% of TGD people.^24^

There was a steady increase in the proportion of ED presentations per year by TGD people during the study period, mirroring trends in other healthcare settings.^25^ Literature exploring the reasons for this is sparse, but increasing visibility, understanding and acceptance of TGD people in society, along with improved knowledge and access to gender‐affirming healthcare, may be plausible explanations.

The majority of ED presentations by our population were of high acuity. Three‐quarters (76.7%) were triaged as categories 1–3. This was much higher than ED population data reported in NSW (40%)^21^ and Australia (57.6%),^26^ even when Australian data were limited to a similar age range (52.3% for patients between 15 and 34 years). This could be due to the higher proportion of mental health presentations, which are often triaged with greater acuity. However, reluctance to attend the ED with minor problems due to fears of discrimination or mistreatment may also contribute.^11^ These fears may lead to delays in seeking medical care for more serious problems, leading to more advanced and severe illness. Alternatively, TGD people may feel more comfortable attending their GP for minor issues, and the ATS category distribution may reflect more appropriate ED use.

Hospital admission was higher in our population (25.5%) compared to Australian ED population data of a similar age range (19.2% among people between 15 and 34 years),^26^ which may reflect more serious illness. The proportion of our TGD patients that did not wait (4.2%) was lower than Australian ED population data (6.7% for 15–34 years).^26^ Prolonged ED waiting time is an important factor for patients that did not wait^27^ and our population may have been assessed more rapidly due to their higher acuity. In contrast, our TGD patients had a higher proportion of leaving prior to treatment completion (8.7%) compared to Australian data (3.5% for all ages).^26^ A lack of cultural safety and exposure to negative healthcare experiences, such as transphobic discrimination and unnuanced communication or examination by healthcare workers in ED settings,^13^ may contribute to TGD patients leaving prior to treatment completion.

Mental health problems were higher compared to studies of the general ED population.^21^, ^26^ Suicidal ideation was the most common ED presenting problem, and mental health complaints accounted for six of the top 10 presenting problems in our patients. In contrast, a NSW ED population study reported no mental health complaints among the top 10 ED presenting problems for 20–39 year‐olds.^21^ Mental health was also the most common ED diagnostic category among our patients, and this was nearly 10‐fold higher compared to the aforementioned study using identical diagnostic categories (29.4% *vs* 3.0%).^21^ These elevated rates were similar to two ED studies of TGD people in the USA (28.7% and 40%).^15^, ^17^ Disparities in ED mental health presentations may be theorised as a consequence of chronic exposure to high levels of minority stress, though further research is needed.

A substantial proportion of our patients had inconsistencies in their gender or name registered in the eMR. Mismatches may arise due to failure of patient registration forms and the eMR to recognise and include TGD identities, particularly non‐binary identities^28^; fear of disclosing one's TGD identity during registration and triage; and reliance on Medicare information, which can be difficult for patients to update. Misgendering and/or deadnaming in the clinician‐controlled body of the discharge letter occurred in 22.6% of generated letters. This may reflect insufficient education of healthcare staff on the importance and nuanced use of affirming language, or reliance on the auto‐populated demographic details registered in the eMR, which were often mismatched. These hypotheses, however, require further exploration. TGD people may experience significant distress through non‐affirming communication, which may contribute to healthcare dissatisfaction and reluctance to seek future care.^12^

The findings of the present study provide insights into the patient and presentation characteristics of TGD people attending the ED and present several opportunities to improve the care of this group. First, the identification of TGD people in healthcare settings, including the ED, is challenging. This may contribute to the dearth of existing literature and the limitations experienced by this and other studies. Ideally, both the sex assigned at birth and current gender identity (the ‘two‐step method’)^29^ should be collected during patient registration to ensure the proper recognition of TGD individuals, enabling the provision of targeted, culturally sensitive healthcare. Second, the eMR should be updated to allow for easier modification of a patient's gender and name and to include options that better describe TGD identities. This change will help reduce mismatched details and prevent accidental misgendering and deadnaming by clinicians, both in person and in discharge letters. Third, education and training of healthcare staff are required to improve culturally sensitive care. Clinicians should be made aware that TGD individuals may present with higher acuity conditions, particularly mental health problems, and have higher rates of leaving prior to treatment completion. Emphasising affirming language may help reduce misgendering and deadnaming in discharge letters, particularly when mismatches in patient details registered in eMR are common.

### Limitations

This was a retrospective, descriptive study conducted at a single ED, situated in an area containing a large community of LGBTIQ+ people. Therefore, the higher proportion of ED presentations by TGD people in the present study compared to Stroumsa *et al*.^15^ likely reflects greater local community numbers rather than improved identification by this methodology. The number of TGD people identified by the present study likely greatly underestimates the true number of TGD people presenting to the ED. Utilising the triage notes for the identification of TGD people is suboptimal, as these are brief, inconsistently documented, and may not capture TGD people if they conceal their identity during triage. Additionally, the keyword search may not include all terms used to describe TGD people in triage notes. Alternative methods, such as searching other clinical notes or the entire clinical record, were either unavailable or excessively resource‐intensive. These limitations may be addressed through improved collection of gender identity information and translation of this data into the eMR.

Sampling bias was a possibility, as a patient's TGD identity may have appeared more relevant for mental health presentations and less relevant for minor complaints, resulting in differences with triage documentation. A small number of ED frequent presenters also accounted for a substantial proportion of presentations among trans females (46.1%) and trans males (29.6%). Studies of ED frequent presenters demonstrate that those aged 18–39 years most commonly attend for mental health problems and substance use,^30^ potentially skewing the findings of the present study. Extracted data may also be incomplete or scant, as clinicians did not evaluate patients or document their findings in a standardised manner. Consequently, under‐reporting of medical, mental health and substance use histories may have occurred. Finally, due to resource limitations, our study was limited to 5 years.

## Conclusion

TGD people represent a small but growing proportion of presentations to our ED. They often presented to our ED with acute mental health problems, had higher acuity presentations, and a greater proportion left prior to treatment completion compared to studies of the broader ED population. Inconsistencies in the patients' gender and name registered in the eMR and misgendering and/or deadnaming on discharge letters were common. More must be done to improve culturally sensitive and inclusive healthcare for TGD people, including improvements in the patient registration process, eMR, and education and training of healthcare staff.

### Competing interests

None declared.

## Supporting information


**Appendix S1.** Variables extracted from Discern Analytics 2.0.
**Appendix S2.** Search terms.

## Data Availability

The data that support the findings of this study are available on request from the corresponding author. The data are not publicly available due to privacy or ethical restrictions.
