# Supplementary material for: Emergency department presentations by trans and gender diverse people in Sydney, Australia: Retrospective case series
Source: Emerg Med Australas. 2025 Mar 19;37(2):e70031. doi: 10.1111/1742-6723.70031 (PMC11920772; doi:10.1111/1742-6723.70031)
Supplement: Supplementary file 1 — Appendix S1. Variables extracted from Discern Analytics 2.0. Appendix S2. Search terms. [file EMM-37-0-s001.docx]

**Appendix**

**Appendix S1: Variables extracted from Discern Analytics 2.0**

Tracking ID

Encounter ID

Arrive date and time

Arrive day

Medical record number (MRN)

Visit ID

Encounter type

Patient name

Gender

Date of birth

Age

ED source of referral

Mode of arrival

Triage category

Presenting problem

Triage comment

Admitting service

Admit ward

ED checkout date and time

Discharge diagnosis

Discharge code

Discharge disposition

ED length of stay

**Appendix S2: Search terms**

| **1. ED triage note** |
| --- |
| Gender  Gender_^a^  Gender.  Gender-  Gender,  Trans_  Trans-  Trans,  Trans.  Trans)  Transgender  Transman  Transmale  Transboy  Transwoman  Transfemale  Transgirl  TransM  TransF  Transfem*  Transmasc*  TG_  TG.  TG-  TG,  TGD  TGNB  TGNC  Binary  Non-binary  Nonbinary  Non_binary  NB_Trans  AMAB  AFAB  MF_Trans  MF-Trans  FM_Trans  FM-Trans  MTF  M_T_F  M-F  M_–_F  MtoF  M_to_F  M2F  M_2_F  FTM  F_T_M  F-M  F_–_M  FtoM  F_to_M  F2M  F_2_M  M>F  M_>_F  M->F  M_->_F  M-->F  M_-->_F  M--->F  M_--->_F  F>M  F_>_M  F->M  F_->_M  F-->M  F_-->_M  F--->M  F_--->_M  Male*female  Male_Female  Male-Female  Male_-_Female  Maletofemale  Male_to_female  Male2female  Male_2_female  Female*male  Female_Male  Female-Male  Female_-_Male  Femaletomale  Female_to_male  Female2male  Female_2_male  Feminine  Masculine  Pronoun*^b^  He/him  He_/_him  Him/he  Him_/_he  He_him  Him_he  She/her  She_/_her  Her/she  Her_/_she  She_her  Her_she  They/them  They_/_them  Them/they  Them_/_they  They_them  Them_they  Name  Prefer*  Identi*  Diverse  Dysphoria  Nonconform*  Non-conform*  Non_conform*  Transition*  Hormone  HRT  Estrogen  Testosterone  Blockers  **Offensive terminology**  Transsexual  Tranny  Shemale  She-male  She_-_male  She_male  She-he  She_-_he  She_he  She2he  She_2_he  She>he  She_>_he  She->he  She_->_he  She-->he  She_-->_he  She--->he  She_--->_he  He-she  He_-_she  He_she  He2she  He_2_she  He>she  He_>_she  He->she  He_->_she  He-->she  He_-->_she  He--->she  He_--->_she  **Misspellings**  Pronous  Gender  Disphoria  Dysforia  Oestrogen  Eostrogen  Transistioning  Hormon  dymorphia  Tansgender |
| **1.2 Gender** |
| Indeterminate  Other  Unknown |
| **1.3 Name** |
| Prefer*  Prefers  Preferred |

**Footnotes**

^a^“_” indicates a space

^b^wildcard search
